# Extended Reality–Based Mobile App Solutions for the Therapy of Children With Autism Spectrum Disorders: Systematic Literature Review

**DOI:** 10.2196/49906

**Published:** 2024-02-19

**Authors:** Marian-Vladut Toma, Cristina Elena Turcu, Corneliu Octavian Turcu, Sorin Vlad, Doru Eugen Tiliute, Paul Pascu

**Affiliations:** 1 Faculty of Economics, Administration and Business “Stefan cel Mare” University of Suceava Suceava Romania; 2 Faculty of Electrical Engineering and Computer Science University of Suceava Suceava Romania

**Keywords:** autism, autistic, autism spectrum disorder, ASD, virtual reality, augmented reality, extended reality, mixed reality, mobile app, children, preschool, mobile phone

## Abstract

**Background:**

The increasing prevalence of autism spectrum disorder (ASD) has driven research interest on the therapy of individuals with autism, especially children, as early diagnosis and appropriate treatment can lead to improvement in the condition. With the widespread availability of virtual reality, augmented reality (AR), and mixed reality technologies to the public and the increasing popularity of mobile devices, the interest in the use of applications and technologies to provide support for the therapy of children with autism is growing.

**Objective:**

This study aims to describe the literature on the potential of virtual reality, AR, and mixed reality technologies in the context of therapy for children with ASD. We propose to investigate and analyze the temporal distribution of relevant papers, identify the target audience for studies related to extended reality apps in ASD therapy, examine the technologies used in the development of these apps, assess the skills targeted for improvement in primary studies, explore the purposes of the proposed solutions, and summarize the results obtained from their application.

**Methods:**

For the systematic literature review, 6 research questions were defined in the first phase, after which 5 international databases (Web of Science, Scopus, ScienceDirect, IEEE Xplore Digital Library, and ACM Digital Library) were searched using specific search strings. Results were centralized, filtered, and processed applying eligibility criteria and using the PRISMA (Preferred Reporting Items for Systematic Reviews and Meta-Analyses) guidelines. The results were refined using a technical and IT-oriented approach. The quality criteria assessed whether the research addressed ASDs, focused on children’s therapy, involved targeted technologies, deployed solutions on mobile devices, and produced results relevant to our study.

**Results:**

In the first step, 179 publications were identified in Zotero reference manager software (Corporation for Digital Scholarship). After excluding articles that did not meet the eligibility or quality assessment criteria, 28 publications were finalized. The analysis revealed an increase in publications related to apps for children with autism starting in 2015 and peaking in 2019. Most studies (22/28, 79%) focused on mobile AR solutions for Android devices, which were developed using the Unity 3D platform and the Vuforia engine. Although 68% (19/28) of these apps were tested with children, 32% (9/28) were tested exclusively by developers. More than half (15/28, 54%) of the studies used interviews as an evaluation method, yielding mostly favorable although preliminary results, indicating the need for more extensive testing.

**Conclusions:**

The findings reported in the studies highlight the fact that these technologies are appropriate for the therapy of children with ASD. Several studies showed a distinct trend toward the use of AR technology as an educational tool for people with ASD. This trend entails multidisciplinary cooperation and an integrated research approach, with an emphasis on comprehensive empirical evaluations and technology ethics.

## Introduction

### Background

In recent years, there has been increased interest in using technology to address the unique challenges faced by individuals with autism spectrum disorder (ASD). Among the various technological approaches, extended reality (XR), which includes augmented reality (AR) and virtual reality (VR), has emerged as a promising solution for intervention and therapy in children with ASD. XR offers the potential to create immersive and engaging environments that can address the specific needs of individuals on the autism spectrum, assisting them with communication, social interaction, and skill development. As a result, researchers and practitioners have explored the development of XR-based mobile apps tailored to the therapy of children with ASD.

However, the rapid growth in this field has spawned a multitude of XR-based mobile app solutions, each claiming unique benefits and features. With this proliferation of interventions, it is important to comprehensively assess the current landscape of XR-based mobile apps for the therapy of children with ASD, not only to strengthen existing knowledge in the field but also to provide critical insights into the research field.

In light of these considerations, this systematic literature review aimed to explore and assess the current status of XR-based mobile app solutions for the therapy of children with ASD. By synthesizing evidence from existing studies, this review aimed to provide an updated overview of the field, identify research gaps, and provide valuable insights. In this endeavor, this review aimed to contribute to the advancement of knowledge and practice in the field of XR-based interventions for ASD therapy, ultimately aiming to improve the quality of life of children on the autism spectrum and their caregivers.

ASD is a neurological condition that has a significant negative impact on a person’s social, verbal, and physical abilities. Researchers claim that ASD is typically discovered around the third year of life [1], but it can be identified and diagnosed as early as the age of 18 months [2]. According to a study from 2022, a total of 1% of infants have ASD [3]. On the basis of studies conducted over the past 50 years, the World Health Organization predicts a global increase in the prevalence of ASD [3].

Researchers consider 3 techniques that could be used to facilitate the evaluation of this condition’s prevalence: providing diagnostic tools, enhancing diagnostic standards, and increasing public awareness of ASDs [4].

On the basis of each individual’s verbal IQ and level of language delay, the diagnosis assesses the severity of the disorder (mild, moderate, or severe) [5], which can estimate the extent to which daily life is affected. Many people with ASD are timid, have difficulty communicating, or experience anxiety when engaging in casual conversation. Despite their communication and social skill deficits, individuals with ASD have demonstrated a preference for technology [6]. Furthermore, the use of technology in behavioral therapy for people with ASD has the additional advantage of being cost-effective in terms of both caregiver and treatment facility expenses [7]. In addition, various studies have shown that individuals with ASD respond better to visual stimuli than to other sensory stimuli [8]. The findings of these studies have led to various applications of technology in digital behavioral treatment.

### XR, AR, VR, and Mixed Reality

As evidenced, the evolution of IT has accelerated in recent years. According to the study by Abad-Segura et al [9], rapid technological advancements have caused a significant and positive shift in how people view modern living.

**Figure 1:**
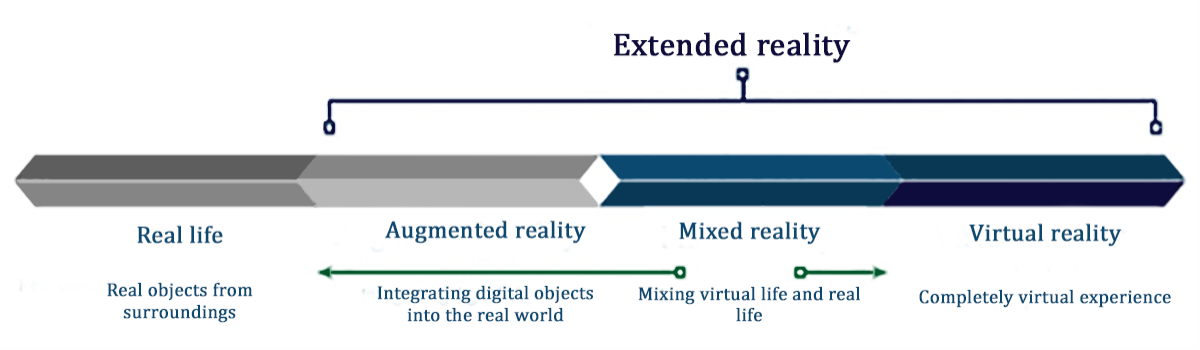
The extended reality concept [10].

The relatively new term XR refers to the entire spectrum from AR to VR, including mixed reality (MR; Figure 1 [10]).

AR is a technology that enables real-time interaction and integration of 3D virtual models into the physical world [11]. Although the first portable AR system was developed in 2003 [12], AR did not acquire widespread acceptance and public awareness until the release of the mobile game Pokémon GO [13] in 2016. Despite the various implementation challenges, AR has many potential applications. In addition to applications in specific domains such as industry, construction, or medicine, as well as in advertising and commerce, education, and gaming [14], AR can be used by a broader audience for everyday tasks such as finding information about nearby points of interest, navigation, and assistance while following a route [14]. Most of these apps are now accessible owing to advances in mobile device technology and the spread of smart mobile phones. AR facilitates behavioral therapy by enhancing the experiences and abilities of people with ASD and establishing an integrated learning environment that enables the visualization of educational materials in 3D and engaging manipulation of real-world objects [15]. By generating “physical” structures to improve specific skills, AR fosters the imagination of patients with ASD without impairing it [16,17]. Moreover, AR can be used to create more engaging and appealing user interfaces, thereby eliminating the need for conventional input devices such as a keyboard and mouse [18]. AR technology is typically accessed using various devices and platforms. Among the widely used platforms and tools for developing AR apps are Unity, Unreal Engine, ARCore, and HP Reveal.

As described in the study by Azuma [11], VR is a computer-generated environment that simulates real-life scenarios, creating an immersive and interactive experience. This means that the users are placed in a completely virtual world, which can be similar to or different from the real one. This technology requires specialized equipment, such as VR headsets or glasses to enable users to see and interact with the virtual environment.

As seen in the studies by Bursali and Yilmaz [19] and El-Jarn and Southern [10], MR is situated between AR and VR, integrating the 2 technologies to provide the user with a unique and captivating experience in real time. It can be difficult to precisely define the limits of MR as they depend on the devices and equipment used as well as the extent to which VR or AR is incorporated into the final product. A model describing the integration of digital objects from the physical world into the virtual world is shown in the study by Milgram and Kishino [20], which also presented a taxonomy for MR, stating that it can be defined as a part of the human-computer interface field, which integrates VR and AR elements to create an environment in which virtual and real objects coexist and interact.

To be used, technologies from the XR spectrum require specific hardware with an optical sensor [19]. In addition, well-known technology companies such as Google, Facebook, Apple, Amazon, and Microsoft have significantly contributed to the development of AR tools and services [21,22], including handheld devices; holographic screens (Microsoft HoloLens); and heads-up displays, which are mainly designed for MR, tablets, and mobile devices (smartphones).

The development of collaborative XR, which enables simultaneous communication and collaboration among multiple users, is one of the research trends in the field of XR [23].

Given the benefits that AR, VR, and MR can offer as a new mode of human-computer interaction and the fact that these technologies are becoming ubiquitous and part of our daily lives, this systematic review aimed to describe how these technologies can be used in the therapy of children with ASDs.

## Methods

### Overview

According to Kitchenham [24], a systematic literature review is a method for identifying, evaluating, and interpreting all available research relevant to a field of study as well as answering specific research questions (RQs). We conducted this research following the PRISMA (Preferred Reporting Items for Systematic Reviews and Meta-Analyses) guidelines [25] and the recommendations suggested by Kitchenham [24].

To conduct this literature review, several well-known scientific databases were queried, and publications containing relevant information for our analysis were filtered. We defined RQs and provided answers to each of them, thus achieving our proposed objective.

### Search Strategy

According to the considered methodology, the following 7 RQs were formulated. These questions consider aspects relevant to the understanding of concepts important to this study:

1. What is the papers’ distribution over time? (RQ 1)
2. What category of people are the studies aimed at? (RQ 2)
3. Which technologies are used with XR or any of its subdivisions to develop apps for ASD therapy? (RQ 3)
4. What skills were targeted for improvement in primary studies? (RQ 4)
5. What are the purposes for which the proposed solutions were used? (RQ 5)
6. What are the results obtained using the proposed solutions? (RQ 6)

To study the literature and answer the aforementioned questions, we searched for scientific publications using various academic research databases. Our study primarily focused on the technical aspects of mobile app solutions using XR for autism therapy. To comprehensively cover our research domain, we chose to use multidisciplinary scientific databases—Scopus, ScienceDirect, and Web of Science—along with 2 databases particularly relevant to computer science, namely, IEEE Xplore Digital Library and ACM Digital Library. From these sources, we only considered publications that were relevant in computer science–related categories, such as technology, engineering, and computer science, excluding categories related to medicine, chemistry, or neurosciences considering that the RQs were focused not only on the available apps but also on their technical details. The functionalities, the technologies used, and the entire process of their development also constituted an objective. Thus, the approach from a technical point of view and the development of these apps were followed. This was done using the results refinement interface available in the aforementioned databases. Initially, to view and analyze the results of queries conducted using the considered search strings, the search was not limited to a particular time.

Given the topic of this study, we aimed to query scientific databases so that the resulting list of publications would meet the following criteria:

1. Reference to ASDs
2. Consideration of one of the technologies that are part of the concept of XR (VR, AR, or MR)
3. Addressing mobile apps
4. Aim to develop solutions for the therapy of children

The literature was searched using keywords relevant to achieving the proposed objectives: *autism*, *autistic*, *ASD*, *virtual reality*, *augmented reality*, *extended reality*, *mixed reality*, *mobile application*, and *children*.

Following the analysis of these keywords, the query process was extended by including the following terms: Autis*, *VR*, *AR*, *MR*, *XR*, Mobile app*, Smartphone app*, Child*, Infan*, Toddler*, Preschool*, Kid*, and *Juvenile*. In the aforementioned list, an asterisk stands for any number of characters at the end of the current string (eg, Preschool* refers to *Preschool*, *Preschooler*, and *Preschoolers*).

### Information Sources

Depending on the search options available in each database considered, specific search strings were defined for querying the databases (Table 1). These query strings were defined using advanced search functions and appropriate operators. The search of Web of Science and Scopus publications was performed by title, abstract, and keywords, and IEEE Xplore Digital Library, ScienceDirect, and ACM Digital Library were searched using a general search. The queries were executed on December 18, 2022.

**Table 1:** The search strings used for querying the databases (N=219).

Item	Database	Search string	Returned results, n (%)
1	Web of Science	(TS=(Autis*) OR TS=(ASD)) AND (TS=(virtual reality OR VR) OR TS=(augmented reality OR AR) OR TS=(mixed reality OR MR) OR TS=(extended reality OR XR)) AND (TS=(mobile app* OR smartphone app*)) AND (TS=(child* OR infan* OR toddler* OR preschool* OR kid* OR juvenile))	45 (20.5)
2	Scopus	(TITLE-ABS-KEY (autis*) OR TITLE-ABS-KEY (asd)) AND (TITLE-ABS-KEY (“virtual reality” OR vr) OR TITLE-ABS-KEY (“augmented reality” OR ar) OR TITLE-ABS-KEY (“mixed reality” OR mr) OR TITLE-ABS-KEY (“extended reality” OR xr)) AND (TITLE-ABS-KEY (“mobile app*” OR “smartphone app*”)) AND (TITLE-ABS-KEY (child* OR infan* OR toddler* OR preschool* OR kid* OR juvenile))	32 (14.6)
3	IEEE Xplore Digital Library	(Autis* OR ASD) AND (Augmented reality OR AR OR Mixed reality OR MR OR Extended Reality OR XR OR Virtual Reality OR VR) AND (Mobile OR Tablet OR Smartphone OR Phone OR Smartglass) AND (App* OR Solution*) AND (child* OR kid* OR infan* OR preschool* OR juvenile OR toddler*)	26 (11.9)
4	ScienceDirect	(“Autism Spectrum Disorder” OR “ASD”) AND (“Augmented reality” OR “Mixed reality” OR “Extended reality”) AND (“App OR Application”) AND (“kids OR children”)	48 (21.9)
5	ACM Digital Library	[[All: “autis*”] OR [All: “asd”]] AND [[All: “augmented reality”] OR [All: “mixed reality”] OR [All: “virtual reality”] OR [All: “extended reality”]] AND [All: “mobile”] AND [[All: “app”] OR [All: application]]	68 (31.1)

### Eligibility Criteria

The papers obtained by querying scientific databases had an interdisciplinary nature. However, our study took a technical and IT-focused approach to mobile app solutions using XR for autism therapy. Therefore, we needed to refine the results by considering inclusion and exclusion criteria. As previously stated, no constraints were imposed on the publication dates of the articles during the search conducted in the scientific databases. Nevertheless, considering the significant progress in mobile device capabilities and their widespread use over the last decade, which have facilitated the development and growth of the global use of XR-based mobile apps for therapeutic purposes, we focused our investigation on the period following 2012 [26,27]. In line with our technical focus on mobile app solutions using XR for the therapy of children with ASD, we refined the search results across the 5 considered databases, prioritizing computer science–related domains. We deliberately excluded categories related to medicine, chemistry, or neurosciences as our RQs focused on both the available apps and their technical details.

Before centralizing the results for analysis, they were refined according to the inclusion and exclusion criteria. The inclusion criteria were as follows:

1. Articles published in English
2. Articles published after 2012

The exclusion criteria were as follows:

1. Book chapters
2. Paper tables of contents
3. Articles published in languages other than English
4. Results on the topics of medicine, chemistry, or neurosciences

After initial processing, the database searches returned the number of results presented in Table 2.

**Table 2:** Results obtained after initial data processing (N=179).

Database	Results, n (%)
Web of Science	43 (24)
Scopus	22 (12.3)
IEEE Xplore Digital Library	25 (14)
ScienceDirect	41 (22.9)
ACM Digital Library	48 (26.8)

### Selection Process

During the selection process, the PRISMA guidelines were considered [25]. These guidelines outline 3 steps: identification (centralizing the results and excluding duplicate publications), screening (review of titles and abstracts and testing eligibility), and inclusion (the publications identified as answering the proposed RQs). The PRISMA 2020 checklist is available in Multimedia Appendix 1. The Zotero reference manager software (Corporation for Digital Scholarship) was used to perform the specified steps. In the first step, 179 publications resulting from the search of the 5 considered databases were imported, after which duplicate publications (n=20, 11.2%) and some conference papers (n=4, 2.2%) were removed. For the next step, 86.6% (155/179) of the publications were considered. To enable author collaboration, the data were imported into Google Sheets. In total, 2 reviewers (M-VT and CET) conducted an independent screening of publications for inclusion based on title and abstract analysis. Studies meeting the eligibility criteria according to both reviewers were then considered for full-text screening. Any disagreements were discussed face-to-face between the reviewers, and a third party was involved to help reach unanimity where necessary. The same process was implemented for the full-text review with the assistance of a third reviewer (SV).

After reviewing the titles and abstracts, a total of 41.9% (65/155) of the publications were excluded as they did not address the proposed topic, focusing either on another condition or on other technologies.

Despite the high quality of the publications, as evidenced by their indexing in prestigious international databases, the analysis included a full text review (where available) of the remaining 58.1% (90/155) of the publications, and the following quality assessment criteria were applied to ensure their relevance to the RQs considered. Articles with no full text accessible were excluded. The following quality criteria (QCs) were applied to 78 publications:

1. Does the research topic address ASDs? (QC 1)
2. Does the study address children’s therapy? (QC 2)
3. Does the study include one of the technologies targeted in this review? (QC 3)
4. Is the solution deployed on a mobile device? (QC 4)
5. Are the results relevant to this review? (QC 5)

Given the nature of the research and its objectives, the 5 QCs that were developed specifically to achieve the goals of the research were used to evaluate the studies’ quality by 2 authors. Each publication was carefully reviewed and assigned a score from 0 to 2 measuring the extent to which it corresponded to the quality assessment criteria and to the subject of this study (0=no; 1=partially; 2=yes). Thus, the maximum score for a paper could be 10. After that, the data were combined for further analysis using Microsoft Excel (Microsoft Corp). After consolidation, a score with a decimal part (eg, 1.5) was rounded up to the nearest integer for better inclusion.

**Figure 2:**
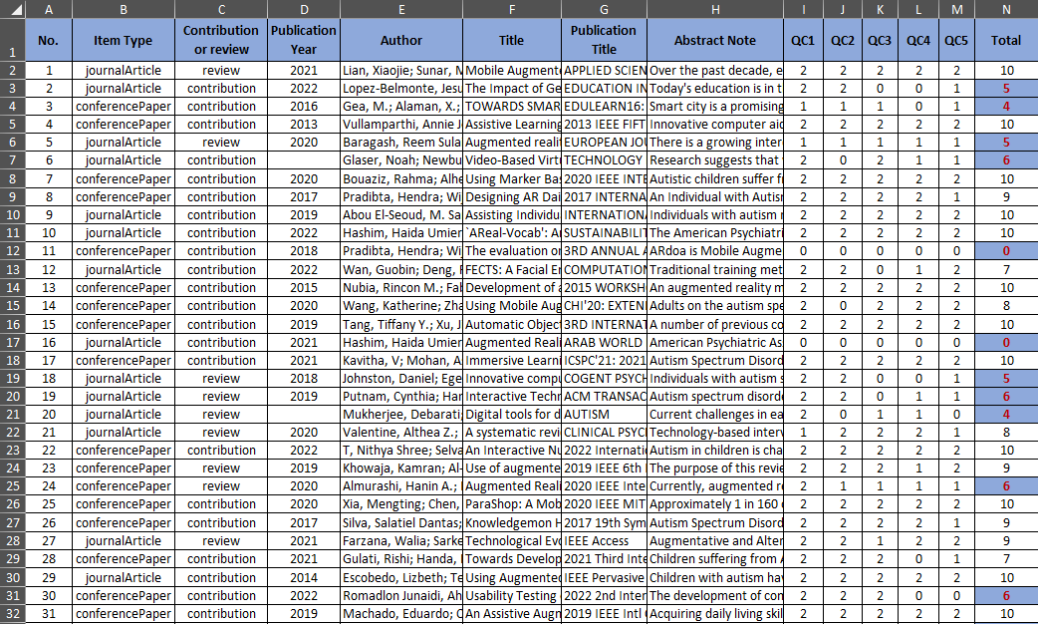
Screenshot of the quality assessment of the papers.

After reviewing the full texts, 3 publications were found to be not written in English, and in other publications, ASD was not addressed (only mentioned), other technologies were addressed, or the proposed solution was not clearly described and the results were inconclusive (see the sample in Figure 2; the entire table is available in Multimedia Appendix 2 [28-104]).

**Figure 3:**
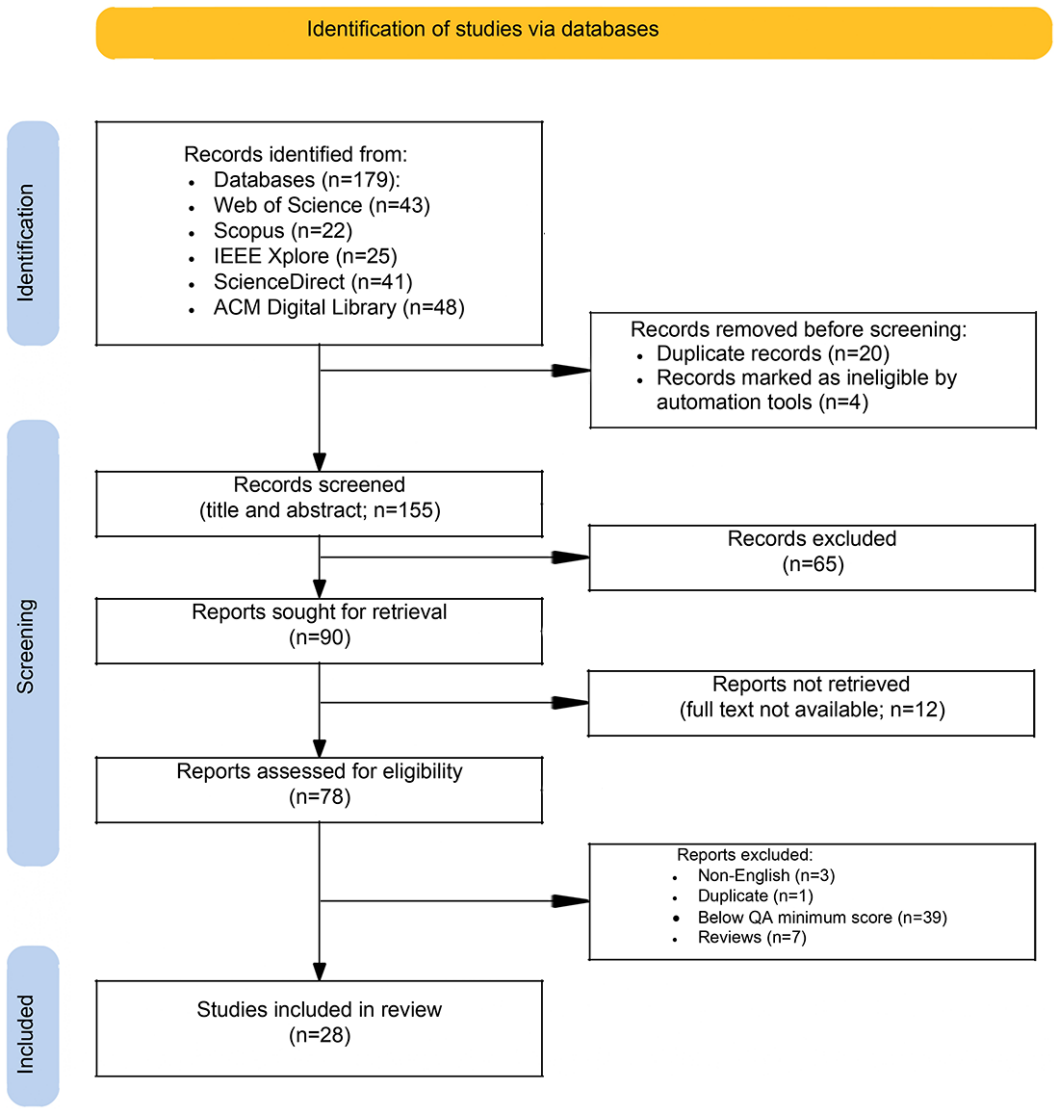
The PRISMA (Preferred Reporting Items for Systematic Reviews and Meta-Analyses) flow diagram. QA: quality assessment.

Following this, only publications with a score of ≥7 were evaluated as they adequately addressed the RQs. The type of publication, whether it was a review or aimed at developing an app, was also noted. Systematic literature review publications were investigated to identify any references that could be added to this study, but they were removed from the list after being reviewed. The entire publication selection process is illustrated in Figure 3.

### Synthesis Methods

The data extraction process was carried out to methodically address the stated RQs. Initially, a single reviewer handled the task of data extraction, leveraging the analytical capabilities of Microsoft Excel to facilitate a structured and organized approach to data collection and analysis. The same tool was used for data organization and representation. Within this app, a comprehensive table was constructed in which the rows were designated to the considered references and data corresponding to individual RQs were entered into separate columns, fostering a systematic representation of the data obtained. Subsequently, to enhance the reliability and validity of the data integration process, a second reviewer performed a verification of the initially extracted data. This encompassing procedure ensured a high degree of accuracy and reduced potential discrepancies, thus guaranteeing the integrity of the data.

## Results

As a result of applying the PRISMA guidelines, a total of 28 publications were considered in this study to address the RQs.

### RQ 1: What Is the Papers’ Distribution Over Time?

**Figure 4:**
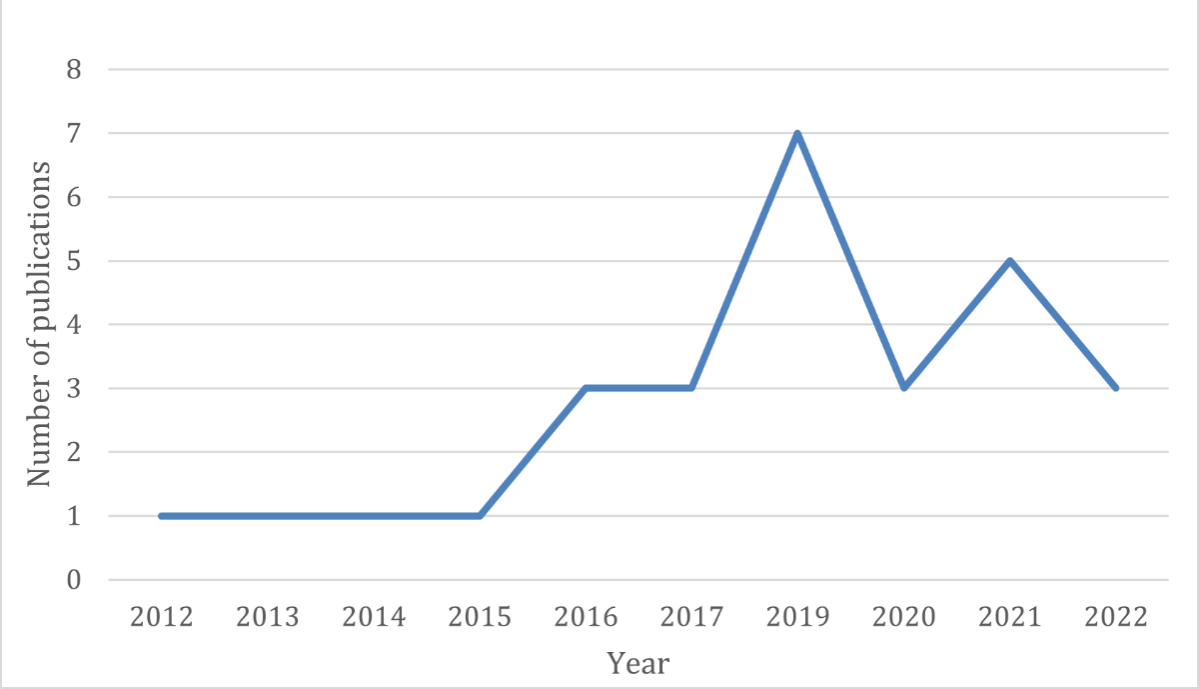
Time distribution of the reviewed publications.

Considering the time range for paper analysis, Figure 4 depicts the time distribution of publications over the period of 2012 to 2022.

Analyzing Figure 4, we can see an increasing trend in the number of publications starting in 2015, with the maximum value being reached in 2019. This denotes an increasingly high interest in these technologies. The number of publications decreased again in 2020, probably because of the pandemic, which limited human interaction and prevented the development and testing of apps dedicated to children with autism.

Regarding the types of publications, more than half (17/28, 61%) were presented at conferences, and 39% (11/28) were articles published in specialized scientific journals.

### RQ 2: What Category of People Are the Studies Aimed at?

The analysis of the considered publications revealed that 43% (12/28) [29,31-33,36,39,43,44,47,50,51,53] stated that they were about children without mentioning the number of participants or their ages. Textbox 1 summarizes the data obtained.

The paper by Xia et al [47] addressed people with autism without mentioning whether they were children or adults, and the study by Wang et al [54] only involved adults but was of interest because the proposed solution can be applied to children as well.

In addition, the studies by Zheng et al [38], Escobedo et al [46], and Voss et al [49] included children both with and without ASD to compare the results and the process recorded in both cases.

**Number and age of the children involved in the studies.**

- Hashim et al [28]: 6 children aged between 5 and 12 y
- Machado et al [29]: children—age not stated
- Tang et al [30]: children aged <4 y and between 4 and 8 y; number not mentioned
- Selvarani et al [31]: children—age not stated
- Abou El-Seoud et al [32]: children—age not stated
- Vullamparthi et al [33]: children—age not stated
- Singh et al [34]: children aged between 9 and 12 y
- Chen et al [35]: 6 teenagers aged between 11 and 13 y
- Tang et al [36]: children—age not mentioned
- Giraud et al [37]: 12 children aged between 5 and 9 y
- Zheng et al [38]: 12 children, 6 with autism spectrum disorder (ASD) and 6 with typical development
- Pradibta and Wijaya [39]: children—age not stated
- Nubia et al [40]: 6 children (5 boys and 1 girl) aged between 3 and 9 y
- Sait et al [41]: 9 children aged between 4 and 12 y
- Wan et al [42]: 10 children aged between 3 and 8 y
- Kavitha et al [43]: children—age not stated
- Silva et al [44]: children—age not stated
- Kalantarian et al [45]: 8 children aged between 6 and 12 y
- Escobedo et al [46]: unknown number of children aged between 8 and 11 y, including 3 children with autism
- Xia et al [47]: mainly people with autism
- Amado et al [48]: children aged between 7 and 9 y; number not indicated
- Voss et al [49]: 20 children with ASD and 20 children without ASD
- Washington et al [50]: 14 families
- Gulati and Handa [51]: children—age not stated
- Escobedo et al [52]: 12 children and 7 teachers
- Bouaziz et al [53]: children—age not stated
- Wang et al [54]: 4 adults, but the system was suitable for children as well
- Gelsomini et al [55]: 5 children (2 with mild ASD, 2 with medium ASD, and 1 with psychomotor retardation)

### RQ 3: What Technologies Are Used With XR or Any of Its Subdivisions to Develop Apps for ASD Therapy?

AR mobile apps for therapy for children with ASD typically used a combination of the following technologies:

1. Mobile devices such as smartphones and tablets equipped with cameras; displays; and sensors such as accelerometers, gyroscopes, and GPS
2. AR software development kits such as ARKit, ARCore, and Vuforia, which provide the tools and framework needed for developing AR apps
3. Graphical and game engines such as Unity and Unreal Engine for 3D model development and creating animations and interactive environments
4. Natural language processing and speech recognition technologies for creating voice-activated AR experiences
5. Computer vision and image-processing techniques for real-time object tracking and recognition of objects, faces, and gestures
6. Machine learning algorithms for customizing the AR experience based on the child’s performance and preferences
7. Cloud computing infrastructure for data storage, management, and analysis of therapy progress

Of the 28 analyzed publications, 22 (79%) addressed a solution from the spectrum of AR implemented on mobile devices such as smartphones owing to their processing power and integrated sensors that make them suitable tools for implementing apps without the need for additional and sophisticated equipment. In addition, the papers by Giraud et al [37], Sait et al [41], Gulati and Handa [51], and Gelsomini et al [55] presented solutions based on VR. Although the articles by Wan et al [42] and Kalantarian et al [45] did not present an AR or VR solution, the methodology addressed and the results obtained show the potential for research in this area. Textbox 2 summarizes information about the technologies and platforms used for app development.

Unity 3D and Vuforia were among the most common platforms used in the development of AR apps for mobile devices, with the Android operating system often mentioned. Several studies (6/28, 21%) [29,38,41,49,51,55] used wearable devices such as Google Glass, Oculus Go, Google Cardboard, Leap motion sensors, and E4 wearable sensors along with the mentioned technologies. Interactive cards were also used as markers to overlay virtual content.

**Technologies and platforms used.**

- Hashim et al [28]: interactive cards, augmented reality, and smartphones
- Machado et al [29]: augmented reality based on smart glasses and Android, web platform, Node.js, eye tracker, sensors, and Amazon Alexa
- Tang et al [30]: augmented reality and Google TensorFlow
- Selvarani et al [31]: interactive cards, augmented reality based on markers, Vuforia, Android smartphone, and Unity 3D
- Abou El-Seoud et al [32]: augmented reality based on markers, smartphones, and the Aurasma framework
- Vullamparthi et al [33]: smartphone, Android, augmented reality, and QR codes
- Singh et al [34]: desktop app and augmented reality
- Chen et al [35]: Vuforia and smartphone or tablet PC
- Tang et al [36]: Google TensorFlow, augmented reality, and smartphone or PC
- Giraud et al [37]: virtual reality (VR) and Unity 3D
- Zheng et al [38]: augmented reality, Microsoft Kinect, and portable E4 sensor
- Pradibta and Wijaya [39]: interactive cards, augmented reality, Android smartphone, and Adobe for animation and graphic illustration
- Nubia et al [40]: augmented reality, Android tablet PC, Unity 3D, Vuforia, and Blender
- Sait et al [41]: VR, Unity 3D, and VR glasses (Oculus Go)
- Wan et al [42]: system that can be implemented on a PC, smartphones or robots; no use of augmented reality or VR
- Kavitha et al [43]: augmented reality, Android smartphone, Vuforia, and ARCore
- Silva et al [44]: augmented reality, smartphone or tablet PC, and Vuforia
- Kalantarian et al [45]: Android smartphone; no VR or augmented reality
- Escobedo et al [46]: augmented reality and Android smartphone
- Xia et al [47]: augmented reality, Android or iOS smartphone, React, Node.js, and Python for object recognition
- Amado et al [48]: augmented reality, Vuforia, Unity 3D, Android smartphone, Balsamiq Mockups 3, and Tinkercad
- Voss et al [49]: augmented reality, Android smartphone, and Google Glass
- Washington et al [50]: Google Glass and Android smartphone
- Gulati and Handa [51]: VR, Leap motion sensors, and VR camera
- Escobedo et al [52]: augmented reality, smartphone or tablet PC, PC server, MySQL database, and HTTP
- Bouaziz et al [53]: interactive cards, augmented reality, smartphone, and Vuforia
- Wang et al [54]: augmented reality, tablet PC or smartphone, Unity 3D, and Vuforia
- Gelsomini et al [55]: VR, Google Cardboard, smartphone, and Unity 3D

### RQ 4: What Skills Were Targeted for Improvement in Primary Studies?

Owing to the deficiencies of children with ASD, the aim was to improve some basic skills such as the following:

1. Communication and language development
2. Social interaction and play skills
3. Fine and gross motor skills
4. Emotional regulation and collaborative strategies
5. Cognitive and problem-solving abilities
6. Attention and ability to follow instructions
7. Independence and self-help capabilities

Table 3 presents the number of publications aimed at improving basic skills.

A total of 25% (7/28) of the publications [28,30,33,36,40,49,52] focused on improving communication skills such as English vocabulary learning [28]; word learning using automatic object recognition through an app based on the TensorFlow library that can be used either when connected to the internet or offline [29]; speaking, reading, and associating images using an app that allows for customization of lessons by parents or therapists [33]; and communication and socialization by delivering certain cues through smart glasses [49]. Textbox 3 details the skills targeted in the studies.

**Table 3:** Targeted learning skills (n=28).

Skill	Studies, n (%)
Religious skills	1 (3)
Daily activities, meal preparation, toothbrushing, and eating	3 (11)
Cognitive or attention	2 (7)
Expressing emotions or social skills	5 (18)
Environment adaptation	1 (3)
Motor skills	2 (7)
Task training	1 (3)
General skills	3 (11)
Number learning	1 (3)
Object recognition	2 (7)
Communication or vocabulary	7 (25)

**Skills aimed to be improved.**

- Hashim et al [28]: communication skills; learning English vocabulary, pronunciation, and articulation skills
- Machado et al [29]: daily routine activities (preparing meals)
- Tang et al [30]: word learning and object recognition
- Selvarani et al [31]: number learning
- Abou El-Seoud et al [32]: general skills; the user can choose the augmented reality (AR) content to be displayed
- Vullamparthi et al [33]: speaking abilities, reading, image associations, and activity scheduling
- Singh et al [34]: procedural task fulfillment
- Chen et al [35]: expressing emotions and social abilities
- Tang et al [36]: object recognition and vocabulary learning skills
- Giraud et al [37]: motor and social skills
- Zheng et al [38]: toothbrushing abilities
- Pradibta and Wijaya [39]: religious abilities—prayers
- Nubia et al [40]: communication abilities
- Sait et al [41]: adaptation to a new or unfamiliar environment
- Wan et al [42]: cognitive skills and practicing facial emotions
- Kavitha et al [43]: general skills; the user can choose the AR content to be displayed
- Silva et al [44]: social and general skills
- Kalantarian et al [45]: expressing emotions and social abilities
- Escobedo et al [46]: social skills in real-life situations, building and maintaining social relationships, improving conversational ability, and managing behavior and emotions
- Xia et al [47]: social and self-help abilities (shopping)
- Amado et al [48]: cognitive skills
- Voss et al [49]: social and communication abilities
- Washington et al [50]: expressing emotions
- Gulati and Handa [51]: motor, focusing, and general skills
- Escobedo et al [52]: object recognition
- Bouaziz et al [53]: self-help skills (feeding)
- Wang et al [54]: attention skills
- Gelsomini et al [55]: general skills (attention, concentration, and understanding) and narration

Some studies (3/28, 11%) focused on the development of self-help skills such as preparing meals with the help of smart glasses, receiving real-time information about the steps to follow [29], brushing teeth [38], or eating [53]. By improving the skills aimed at in the studies considered in this review and developing skills that can improve the deficiencies present in children with ASD, the social inclusion of children with ASD was pursued.

### RQ 5: What Are the Purposes for Which the Proposed Solutions Were Used?

Using at least one of the technologies targeted in this review, the solutions presented in these publications were used to assist children with ASD. Developed for use by both therapists and parents at home or in specialized medical centers, these solutions aimed to improve certain fundamental aspects of the lives of children with autism. Textbox 4 presents information related to the reasons for which the apps were developed.

Hashim et al [28] created an app for the development of children’s English vocabulary, which could potentially be used with other languages as well. The solution proposed in the paper by Machado et al [29] used multiple technologies to allow the therapist to model activities using a web platform and provide hints to users via smart glasses. It also works as an attention-monitoring tool via an eye tracker so that activities can be evaluated and improved. The study by Tang et al [30] addressed the problem of communication through automatic object recognition using a smartphone and display of virtual content (object names) on the screen. This app works either when connected to the internet or offline. A similar approach was observed in the study by Selvarani et al [31], in which children could learn numbers by scanning notebooks using their mobile devices, after which the relevant content was displayed on the screen.

**The purpose of the developed apps.**

- Hashim et al [28]: the Areal-Vocab app was developed to help children with autism improve their English vocabulary.
- Machado et al [29]: the aim was to develop an assistive app using augmented reality (AR) based on smart glasses and a visual attention analysis tool to help people with autism in daily tasks by providing complementary information (eg, to pick up the knife and then cut the strawberries). The therapist can model the activity.
- Tang et al [30]: the researchers intended to develop a mobile app for children with autism that could run either connected to the internet or offline that would improve word learning skills by using object recognition.
- Selvarani et al [31]: the aim was to help children with autism learn numbers by scanning an interactive card using the app so that complementary video and audio content is displayed on the screen.
- Abou El-Seoud et al [32]: the aim was to develop a framework to help parents or educators use AR in a personalized way by choosing what type of AR educational content to display over a printed marker representing a familiar cartoon character.
- Vullamparthi et al [33]: a tool was developed that included an interface for parents or educators to scan a QR code and create various lessons and an interface for children. It used the smartphone camera, an Android apk (Android application package), a web page, a database, and Jakarta server pages.
- Singh et al [34]: the paper was a comparative study that aimed to explore the effectiveness of AR in the execution of tasks among less privileged children (who have had minimal interaction with technology), healthy but younger children, and children with autism.
- Chen et al [35]: the researchers developed a Vuforia-based AR app that can be deployed on Android or iOS devices (smartphones or tablets). This app can scan storybooks (with images captured from videos) and overlay relevant content to assist children with autism in expressing and understanding emotions and developing social skills.
- Tang et al [36]: the aim was to develop a tool for children with autism that can recognize objects and display their names.
- Giraud et al [37]: the study aimed to involve children with autism in common actions (moving furniture) by interacting with a virtual character projected on a tactile magnetized surface.
- Zheng et al [38]: the goal was to develop an AR system (Cheerbrush) that could teach children with autism how to brush their teeth considering how important this is to stay healthy and avoid dental procedures. It uses Kinect to capture the user’s movement, a 3D-printed toothbrush to assess brushing skills, a monitor to view the surroundings, and an avatar. It also uses a wristband to assess children’s stress while using the app.
- Pradibta and Wijaya [39]: the aim was to help children with autism learn daily prayers. The goal was to develop an app that contains animated learning materials in the form of daily prayers from the Islamic religion.
- Nubia et al [40]: the aim was to help children with autism communicate better using an app that can identify human-recognizable objects such as animals, fruits, or other common objects and match them with specific sounds.
- Sait et al [41]: the goal was to develop a virtual reality (VR) framework in which the teacher can enter information about the child and prepare scenes that can be watched by a child wearing VR glasses. The main objective was to familiarize children with autism with places such as school, the schoolyard, and the classroom by previously visualizing the environment.
- Wan et al [42]: the aim was to help children with autism recognize, practice, and express emotions such as happiness, sadness, fear, or anger.
- Kavitha et al [43]: the aim was to help children with autism recognize objects or animals by rendering 3D content over certain images.
- Silva et al [44]: the aim was to help reduce the isolation of children with autism by encouraging them to explore the world with the help of an app based on geolocation and AR.
- Kalantarian et al [45]: the goal was to help children with autism learn to express their emotions. Guess what? is an Android mobile app similar to Heads up!, a game in which a parent holds the smartphone with the screen facing the child, the child imitates what they see, and the parent tries to guess the simulated emotion.
- Escobedo et al [46]: the paper describes the design and development of the MOSOCO app, which is a mobile app that provides real-time support and guidance to children with autism in practicing social skills. The app uses AR technology to overlay social hints directly into the child’s real environment, allowing them to practice social skills in real-life situations.
- Xia et al [47]: the app provided step-by-step guidance for people with autism to go shopping by augmenting real shopping scenes using object recognition, barcode reading, and automatic classification.
- Amado et al [48]: the main objective was to develop an AR mobile app to be used by parents of children with autism for their therapy during the pandemic, when human interaction was limited.
- Voss et al [49]: the system described in the paper aimed to help people with autism spectrum disorder improve their social skills by providing discrete real-time social cues via wearable technology. Social cues are provided directly in the wearer’s field of vision using AR technology and are intended to help the wearer navigate social situations and improve their social interactions and communication skills.
- Washington et al [50]: the goal was to develop an app that runs on an Android smartphone (used by a parent) that is connected to a Google Glass device worn by the child. Social cues are delivered to the glasses based on emotions recognized by the mobile app, which also records the session (video only for privacy reasons). The activities are gamelike—catch the smile, guess the emotions, and unstructured activities.
- Gulati and Handa [51]: the aim was to develop a VR game to improve reading, basic math, and spelling. Motor skills are improved by reading gestures and helping coordinate them with the eyes using the Leap motion sensor.
- Escobedo et al [52]: the aim was to create an app that can identify objects that are tagged and display relevant content over them, such as text, 3D models, vibrations, video, or audio, and the user can receive a reward. The main architecture is composed of a module called therapy manager, an ambient notification system, and a tag manager.
- Bouaziz et al [53]: the aim was to develop an app dedicated to children with autism that teaches them how to eat by scanning an interactive card and displaying on top of it a 3D character depicting the targeted skill.
- Wang et al [54]: the aim was to help adults with autism be more focused by performing certain tasks, such as rearranging objects in a scene.
- Gelsomini et al [55]: the aim was to develop a VR mobile app for smartphones that can be used with Google Cardboard, helping children with autism understand activities through storytelling and allowing caregivers to customize the content using a web app, monitor children’s attention, and analyze statistics.

The apps developed in the studies by Abou El-Seoud et al [32] and Vullamparthi et al [33] aimed to go through some lessons that parents and educators could customize by accessing a web platform so that they could choose which type of content to display when the app detected an object in the visual area. The study carried out by Singh et al [34] compared the effects of apps that use AR to perform certain tasks in both children with ASD and children with typical development. Religious activities were also included in one study [39], which presented an app containing animated materials that helped children learn prayers.

Given the fact that children with autism typically experience difficulties adapting to a new environment, Sait et al [41] aimed to develop an app that uses VR, VR glasses, and a web platform in which therapists can enter information about each child and set up custom scenes, such as a classroom, to be viewed virtually and get used to. In addition to the goal of conducting basic activities, Xia et al [47] developed an app to guide individuals with autism with grocery shopping step by step.

### RQ 6: What Are the Results Obtained Using the Proposed Solutions?

Depending on the proposed solution and the objectives of the studies, the results were different, but in general, where the app was tested, encouraging results were obtained, with the remark that these were to be improved and tested more thoroughly. In cases in which the app was not tested with the intended audience but was proven to function, it was deemed to have potential. Textbox 5 summarizes the results obtained in each study.

Upon analyzing the results of the studies included in this review, it was found that only 68% (19/28) of the apps were tested with children with autism, whereas 32% (9/28) were tested only by the developers for functionality purposes. Regarding the methods used to quantify the results, 54% (15/28) of the studies used interviews, and only 14% (4/28) of the studies used an assessment method based on assigning a score according to the degree of skill improvement after using the apps.

**Summary of the results.**

- Hashim et al [28]: children and their parents or educators in the study used the app and reported positive results based on interviews: “Helps listen and understand instructions, helps maintain attention longer, helps with pronunciation and enunciation, helps keep them engaged and interested to learn the vocabulary in depth.”
- Machado et al [29]: the app has great potential considering the fact that smart glasses can very easily transpose the user into the world of augmented reality (AR) and help them by displaying complementary information, as well as giving feedback to the therapist. It has been tested by developers but has not been tested with children with autism, so it does not show quantifiable results.
- Tang et al [30]: the first pilot study was conducted on a university campus with neurotypical children and adults, who provided positive feedback and showed a lot of interest. The second study was conducted in a special education unit involving 2 groups: one with children aged <5 y and one with children aged between 6 and 8 y. It was noticed that the younger children had difficulty using the app, but it was well received by the older children. Positive feedback was also provided by parents and teachers, pointing out that the offline module required improvement.
- Selvarani et al [31]: the app (NUM09) is functional but has not been tested on children with autism with quantifiable results.
- Abou El-Seoud et al [32]: a total of 3 patients with autism, together with their instructors, performed a usability test. According to responses to a questionnaire, the system can improve communication, concentration, and attention and is easy to use.
- Vullamparthi et al [33]: this study developed an Android smartphone app that helps children with autism and their parents or therapists create personalized lessons to improve basic skills such as reading, writing, or picture recognition. A workshop was held, and positive feedback from parents was reported. There are no quantifiable results.
- Singh et al [34]: the main task was to complete a tangram puzzle. In the first stage, the involved children did not solve the puzzle without clues involving AR, but it was reported that solving took longer in the AR training mode. In the first study, children aged 9 to 12 y rated the desktop-based instruction mode as the least preferable, whereas the performance using the AR mode was superior. In the second study, 4 children with autism followed the same procedure but had difficulty using the AR-based solution, resulting in poorer performance on the task.
- Chen et al [35]: the app was tested in a dedicated room equipped with a computer, a 52-inch monitor, and 8 tablets. The therapist showed the children the app and asked them to look at the pictures, answer some questions, and use the tablet to access the AR content by pointing it at the picture with the app running in the background. Positive feedback was reported from the children, who were curious and eager to discover new visual cues, showing interest in the facial expressions, gestures, and related activities of the characters. The children had low scores on the initial assessment, but all 6 scores increased significantly after the app intervention. The most dramatic improvement was in one child, from 30% to 89.5%.
- Tang et al [36]: the app works, but it has not been tested on children with autism with quantifiable results.
- Giraud et al [37]: 12 children with autism spectrum disorder (ASD; including 2 girls) aged between 5 and 9 y participated in the study for a period of 3 mo. In total, 7 of the children showed little conversational language. A 3-stage experiment was conducted (familiarization, moving an object with an agent following the child, and training with an agent that the child follows). Preliminary results were encouraging: one-third of the children completed the training, another third needed device adjustments, and some had difficulty using the system.
- Zheng et al [38]: to evaluate the system, 6 children aged between 3 and 6 y (3 with ASD and 3 without ASD) were involved in an experiment comparing the results. It was noted that all the children were able to complete the training sessions, but the children with ASD were clearly more engaged and interested. After training, the most notable improvements were observed in children with autism. During an interview, both children and parents said that they liked the app and that it helped them improve their toothbrushing skills.
- Pradibta and Wijaya [39]: no proof of testing with children with autism and no quantifiable results.
- Nubia et al [40]: by playing relevant sounds in line with images, the app helped children improve their learning skills compared with traditional methods. A 14% increase in attention and a 9% increase in verbal language were reported.
- Sait et al [41]: the system was used by 9 children with autism who benefited from the help of therapists who guided them in adjusting the Oculus Go headset and using the app (AutiVE). One of the issues was the virtual reality (VR) headset itself and the VR environment, but the website provided had a video explaining them. In total, 8 of the children eventually accepted the device. There were some improvements in learning skills, but no detailed statistics were mentioned.
- Wan et al [42]: the children completed a 20-min training session each day for 4 consecutive days. A total of 6 participants showed improvement in proficiency in operating the system, 5 of 6 completed all tasks, and 4 of 6 showed improvements in expressing emotions. Children aged <5 y found the app difficult and did not perform in a satisfactory manner.
- Kavitha et al [43]: the app works, but it has not been tested with quantifiable results.
- Silva et al [44]: an app similar to Pokémon GO was developed in which users can find “monsters” in certain areas and, by clicking on them, find relevant information. The concept of gamification was used, but the system was not validated with real users with autism.
- Kalantarian et al [45]: the solution was tested with 8 children, all boys, playing up to 5 games in 1 session. In total, 94%, 81%, 92%, and 56% of the emotions were labeled correctly as disgust, neutrality, surprise, and fear, respectively.
- Escobedo et al [46]: the app was evaluated over 7 wk. Interview results revealed that the app was well received by children with autism and their therapists and that it was effective in helping children practice and improve their social skills in real-world situations. The authors reported that users were able to use the app easily and that the AR technology was effective at providing children with real-time support and feedback. The study also showed that the app was well accepted by therapists, who found it a useful tool for their patients’ therapy.
- Xia et al [47]: the app, called ParaShop, was tested by a nonprofit organization that helps people with disabilities. Staff said that the app helped people with autism buy their groceries, but the number of participants or other details were not mentioned.
- Amado et al [48]: a case study was conducted using Google Forms asking parents to answer questions related to their children (eg, age, gender, and whether the parents lived together). Several studies with parents were conducted, and then the app was developed based on their responses and requirements. In the last stage of the case study, 5 questions were posed about the final prototype of the app. The survey revealed that 46.2% of parents were satisfied and 23.1% were very satisfied. Overall, the mobile app received positive feedback from respondents.
- Voss et al [49]: the research entailed a study involving 20 participants with ASD and 20 participants without ASD who used a system called Superpower Glass over a 4-mo period. The results showed that the participants found the social cues useful in situations and improved their social interactions and communication skills. The study also assessed the acceptability and usability of the system, and the results suggest that it was well received by participants and easy to use.
- Washington et al [50]: the app was tested by families, and they reported that it was useful, with some of them recording the sessions and then showing them to the children to see how they behaved for further improvement. Overall, based on interviews, parents reported positive outcomes.
- Gulati and Handa [51]: the concept of gamification was used; it has potential, but it has not been tested in children with autism. To play the game, a dedicated gaming room and specific equipment are required.
- Escobedo et al [52]: the app (Mobis) was tested with 7 teachers caring for 12 children with autism aged between 3 and 8 y. The researchers conducted weekly interviews with the teachers, keeping in mind that only 3 out of 12 children were able to properly pronounce words. The duration of the observation was 54 h. Participants were reported to find Mobis “exciting, useful, and easy to use.” Students improved their motor skills by focusing the camera on the target. Mobis increased the time that students stayed on task by 20% and motivated them to use the app as they were excited to discover new objects in their environment. Selective attention improved by 62%, and sustained attention improved by 45%. Mobis also induced positive emotions and taught behavioral skills such as tolerance.
- Bouaziz et al [53]: no proof of testing with children with autism and no quantifiable results.
- Wang et al [54]: the app was developed for demonstrative purposes only; it has not been tested with quantifiable results.
- Gelsomini et al [55]: the solution (Wildcard) was tested in a special unit with 5 children with autism during 8 individual therapy sessions. Therapists reported improvements in children’s attention and cognitive skills, but the paper only reported qualitative data. Therapists were excited to be able to customize each VR session and noted that patients embraced the app and found it engaging.

## Discussion

### Principal Findings

The analysis revealed an increasing trend in publications starting from 2015, reaching its highest point in 2019 and followed by a decline in 2020, potentially because of the pandemic. Most of the papers (17/28, 61%) were presented at conferences and largely focused on AR solutions (22/28, 79%) for mobile devices to assist children with ASD in enhancing basic skills and fundamental life aspects. Notably, Unity 3D and Vuforia emerged as popular development platforms. Although a substantial percentage of publications (13/28, 47%) did not provide details on participating children, most of the identified participants were aged between 3 and 13 years. Developed for use by both therapists and parents at home or in specialized medical centers, these solutions showed encouraging preliminary results but underscore the necessity for further, more extensive testing, particularly as a significant portion (9/28, 32%) were only developer tested.

### Main Directions of Research

Upon examining the scientific publications included in our study, several main directions for the use of XR to support children with autism can be identified.

One notable topic is the use of AR in the area of language skills and vocabulary learning in children with autism. Researchers in some studies (3/28, 11%) [28,30,43] focused on the development of AR-based mobile apps that facilitate word learning and object recognition through techniques such as deep learning and automatic object recognition.

Another topic addressed in some studies (3/28, 11%) [29,39,53] was the use of smart glasses or wearable devices to support children with autism in social interactions. These devices provide real-time visual cues and information to enhance communication and social interaction skills.

The use of AR occupational therapy and the development of cognitive skills in children with autism were explored in some studies (3/28, 11%) [31,38,46], which proposed AR-based apps to aid children in learning numbers, teeth-brushing skills, or environmental adaptation skills.

Furthermore, it was stated that the apps specifically designed for children with ASD should be tested with a target group of children, and the results should be quantified in a pertinent manner given that a large part of the findings were obtained through interviews.

Personalization and adaptability are other key aspects of developing mobile apps, as shown in the studies by Wan et al [42], Kalantarian et al [45], and Washington et al [50]. These publications addressed the development of personalized systems and apps to maximize the therapeutic and educational benefits for children with autism.

Some studies (3/28, 11%) [32,34,52] also examined the use of AR to provide individual support for people with autism and cognitive impairment. These studies proposed AR-based frameworks and approaches to assist individuals with autism in various activities and tasks, such as training in procedural tasks, perception and recognition of facial emotions, or assistance in real-life situations.

In addition, some studies (3/28, 11%) [35,37,55] investigated the use of AR in the context of education and social skill development. These studies focused on the use of interactive books, serious games, or training apps to support children with autism in understanding and interpreting facial expressions, social cues, and social interactions.

The use of AR in the context of learning in a geographical environment or learning environmental coping skills was addressed in some studies (3/28, 11%) [40,41,47]. These studies proposed AR-based apps to assist children with autism in exploring and learning in a geographical environment or in developing adaptive skills applicable to different situations and contexts.

A relatively small number of studies (6/28, 21%) [34,38,46-48,52] focused on VR-based approaches for apps, indicating a shift toward the adoption of AR owing to its lower cost and greater usability. Using VR, the study by Abou El-Seoud et al [32] evaluated joint action (moving furniture) abilities using a virtual character, and the preliminary results were encouraging, with one-third of the children completing the training despite 7 of them having limited conversational language. Another issue addressed in the study by Tang et al [36] was adaptation to unfamiliar surroundings. Researchers reported improvements in learning abilities but stated that the equipment and VR environment posed the greatest challenges. The concept of gamification was integrated with VR in the study by Escobedo et al [46] to enhance reading, basic mathematics, and spelling. This app required a special room to run. Although this app has great potential, it has not yet been tested in children with ASD. In addition, an app using Google Cardboard and VR was developed to enhance the cognitive and attentional skills of children. Therapists expressed satisfaction with the outcomes as they were able to personalize each session using unique teaching methods.

### User Interaction Perspectives

#### Overview

The interaction of children with ASD with XR devices, such as AR and VR platforms, brings forth a distinct set of considerations. The manner in which children with ASD use these devices can be influenced by their sensory sensitivities, motor skills, cognitive abilities, and preferences. Although the experiences can vary widely, the following are some ways in which children use XR devices and the challenges they may face.

#### Physical Interaction

Children use XR devices by interacting with touch screens, controllers, or wearable components. They may tap, swipe, or perform gestures to navigate through XR environments. However, children with fine motor difficulties may struggle with precise interactions, leading to accidental inputs or difficulties in selecting desired options.

#### Visual Engagement

Children engage visually with the XR content displayed on screens or through headsets. Visual stimuli can capture their attention and spark interest. Nonetheless, those with sensory sensitivities may experience sensory overload or visual discomfort if the content is excessively bright, flashy, or overwhelming.

#### Spatial Awareness

XR experiences often involve spatial interactions such as moving through virtual environments or manipulating virtual objects. Children’s spatial awareness skills can influence their ability to navigate these environments. Some children may find it challenging to grasp the concept of a virtual space, leading to disorientation.

#### Auditory Response

Many XR apps incorporate auditory cues, sound effects, or voice instructions. Children may respond to auditory prompts by vocalizing or reacting physically. However, children who are sensitive to loud or sudden sounds may experience distress in XR environments with intense auditory stimuli.

#### Attention and Engagement

Children’s level of attention and engagement with XR content can vary. Some may become deeply immersed and engaged, whereas others may have difficulty sustaining their attention because of the novelty of the experience or sensory distractions.

#### Preferences and Comfort

Children’s preferences for certain types of interactions or content can influence their engagement. Some children may appreciate exploring virtual worlds, whereas others may prefer more structured or repetitive activities. Ensuring a variety of XR experiences allows for accommodating different preferences.

#### Transition Challenges

Transitioning between the real world and the XR environment can be challenging for some children. They may have trouble understanding that the virtual elements are not physically present or struggle with transitioning back to reality after prolonged XR use.

#### Response Variability

Children with ASD may respond to XR experiences differently across sessions. Factors such as mood, sensory sensitivities, and cognitive states can influence their interactions. Some days, children may be more receptive to XR, whereas on other days, they may be less engaged or overwhelmed.

#### Calibration and Setup

XR devices require proper calibration and setup for optimal interaction. Children may need assistance in adjusting headsets, ensuring proper alignment, or calibrating controllers. Technical difficulties can lead to frustration or disengagement.

### Challenges

The identified challenges are as follows:

1. Individualized learning needs: a prevalent challenge across the studies in this review was catering to the diverse learning preferences and abilities of children with ASD. For instance, Hashim et al [28] faced the task of addressing the specific needs of children with mild ASD. Similarly, the studies by Abou El-Seoud et al [32] and Singh et al [34] addressed the challenge of tailoring their AR experiences to suit varying preferences and capabilities.
2. Transferability and generalization: a common limitation is the transfer of learned skills to real-world scenarios. As seen in the studies by Tang et al [36] and Giraud et al [37], researchers have encountered challenges in translating acquired skills into practical applications. In addition, studies such as those by Chen et al [35] and Kavitha et al [43] noted limitations in transferring learned skills beyond the AR context, possibly owing to the variations in real-world stimuli.
3. Technical feasibility and personalized support: technical feasibility and ongoing support emerged as challenges in some studies [29,38]. Maintaining the functionality of AR-based smart glasses and ensuring accurate real-time feedback for toothbrushing techniques required continuous technical support.
4. Sensory overload and individualization: sensory sensitivities and the need for individualized solutions were prominent challenges. Sait et al [41] encountered the challenge of designing virtual environments that cater to sensory sensitivities, whereas studies such as the one by Voss et al [49] highlighted the importance of unobtrusive cue presentation in wearables for children with ASD.
5. Cognitive adaptation and user adoption: cognitive adaptation and user adoption challenges were evident in some studies [48,52]. Designing tasks that effectively target cognitive skills and maintaining user engagement over time were key considerations.
6. Ethical implications of data handling: as AR interventions involve interactions and data collection, ethical considerations are paramount. The study by Wan et al [42] delved into recognizing facial expressions, which raises ethical concerns related to data privacy and security. Ensuring that data-handling protocols adhere to ethical standards becomes crucial, underscoring the need to protect sensitive user information while deriving meaningful insights from the interactions.
7. Cross-cultural adaptation and applicability: given the diversity of cultures and languages, ensuring the cross-cultural adaptation and applicability of AR interventions becomes a notable challenge. The study by Wang et al [54], which explored mobile AR for attention improvement in adults with ASD, highlights the importance of adapting interventions to diverse cultural contexts. This challenge emphasizes the need for cultural sensitivity and the localization of content to ensure that interventions are universally accessible and effective.
8. Long-term impact measurement: measuring the long-term impact of AR interventions and tracking the progress of children over time poses significant challenges, as pointed out in the study by Escobedo et al [46], which emphasized the importance of assessing the sustained effects of interventions beyond short-term interactions. This challenge underscores the necessity of devising reliable methodologies for gauging the lasting benefits of AR interventions and understanding how these interventions contribute to the developmental trajectory of children with ASD.

In the realm of AR apps for children with ASD, studies have striven to engage children through various interaction modes while tackling shared challenges. The diverse engagement strategies and the collective endeavor to overcome common limitations underscore the continuous efforts to create meaningful and effective AR-based interventions for this unique demographic.

Research in this area demonstrates an interdisciplinary approach involving collaboration among specialists in education, IT, and mental health. This is illustrated by the diversity of authors and publications included in this review, suggesting that integrating AR, VR, and MR into ASD pedagogy requires a comprehensive approach that considers multiple aspects—from technology design to educational and mental health psychology. It was also specified that the apps aimed at children with ASD should be tested with a target group of children and that the results should be quantified in a more relevant manner given that a large part of the reported results was obtained only through interviews. The analysis of the studies indicates a trend in research toward the use of diverse and innovative study methods, such as using both quantitative and qualitative methods to investigate the impact of AR, VR, and MR on people with ASD.

Furthermore, the analyzed publications suggest that the development and implementation of AR-, VR-, and MR-based technologies extend beyond academia or research, involving partnerships with the private sector and local communities. This demonstrates the awareness of the need to transfer research findings into practice to have a direct impact on people with autism.

### Limitations

Considering the publications reviewed in this study, several limitations were identified regarding the development and testing of AR-, VR-, and MR-based mobile apps:

1. Sample size: some studies involved small samples of participants, which may have limited the generalizability of their results to a larger population of children with autism. The involvement of a limited number of participants in many studies can be attributed to the unique characteristics of the target population—children with ASD. The diversity in ASD manifestation, severity, and individualized needs necessitates careful participant selection. Moreover, recruitment challenges, ethical considerations, and the resource-intensive nature of working with children with ASD contribute to the small sample sizes. However, this limitation was often acknowledged in the papers, along with the understanding that the findings may not be easily generalizable to the broader population with ASD.
2. Study duration: the duration of the studies included in this review varied from short testing sessions to several weeks or months. The short duration of many studies was due to practical constraints and the inherent complexities of conducting research involving children with ASD. Longitudinal studies involving children with ASD can present challenges in terms of participant retention, compliance, and data collection consistency over extended periods. In addition, the rapid pace of technological advancements may affect the relevance of the findings if studies are conducted over prolonged durations. However, the researchers did recognize the limitations imposed by short study durations and provided justifications for the chosen time frames.
3. Diversity of diagnosis and level of functioning: autism is a disorder with a wide variety of symptoms and levels of functioning, which adds complexity and variability to the research.
4. Standardized outcome assessment: some studies did not use standardized outcome assessment tools, which may have affected the comparability and validity of the obtained results.
5. Availability and accessibility of technology: although the presented studies demonstrate the potential of AR technology to support children with autism, it is important to consider the availability and accessibility of this technology in real-world settings. The cost, infrastructure, and availability of AR devices and apps may be limiting factors in the widespread adoption of this technology.

The relatively small sample sizes and short durations commonly observed in many studies involving children with ASD and XR interventions are notable aspects of the research landscape. Although solutions to these issues were not always addressed in the papers, they remain ongoing areas of consideration for researchers in the field. In the selected papers, although some discussions and considerations regarding the challenges of small sample sizes and short study durations were present, comprehensive solutions were not elaborated on. The researchers often acknowledged these limitations and offered potential insights or recommendations, but definitive solutions were not always a primary focus of the papers, the primary focus being on the technical details and potential outcomes of their approach.

In addition, this study itself has several limitations, which should be considered for further research:

1. Limited number of databases queried: despite using comprehensive search strategies, it is possible that some relevant studies were omitted because they were published in nonindexed or less accessible sources.
2. Field evolution: this field of study is rapidly evolving, and new research may have been published since the literature search was conducted. Consequently, this review may not capture the most recent evidence and emerging trends in the field.
3. No distribution of publication authors: this review did not present information on the regional distribution of authors or the origin of the apps and systems, disregarding the influence of cultural differences on the development of these types of apps.
4. Lack of security analysis: this study did not analyze the security issues associated with the proposed solutions.
5. Absence of cost information: no information regarding the cost of the presented solutions could be identified.

### Conclusions

This study aimed to conduct a systematic review of the specialized scientific literature in terms of applications, devices, and technologies relevant to the development of AR-, VR-, and MR-based mobile apps dedicated to the therapy of children with ASDs, an objective that was successfully achieved. At the beginning of this paper, the general concept of ASD was presented, after which the RQs and inclusion and exclusion criteria were defined and the results of applying the PRISMA guidelines for the selection of publications to be reviewed were reported. The answers to the RQs were discussed. At the end of the paper, the limitations of the research were presented.

Although the concepts of AR, VR, and MR are not entirely new, their use in the development of therapeutic apps for children with autism has only recently gained popularity. The findings documented in various publications indexed in 5 scientific databases emphasize the suitability of these technologies for such therapy, thereby warranting further in-depth research and the future development of apps based on these technologies. The studies indicated a clear trend toward the use of AR, VR, and MR technologies as a pedagogical tool for people with ASD. This trend involves multidisciplinary collaborations and an integrated approach to research, with a focus on empirical evaluations and ethics regarding the use of technologies. As the field advances, it is essential that research and practice continue to be guided by a balanced and integrated approach that considers both the technological possibilities and the needs and rights of individuals with ASD. However, there are still many issues that require further exploration and research.

Moreover, the publications studied illustrate a wide range of research areas related to the use of AR, VR, and MR in the context of ASD, as well as a variety of methodological and theoretical approaches adopted by the researchers. This suggests that the field is in a phase of rapid growth and diversification, with a wealth of opportunities for future research and development.

## Appendix

Multimedia Appendix 1 PRISMA (Preferred Reporting Items for Systematic Reviews and Meta-Analyses) 2020 checklist.

Multimedia Appendix 2 Quality assessment of the papers.

## Abbreviations

AR: augmented reality
ASD: autism spectrum disorder
MR: mixed reality
PRISMA: Preferred Reporting Items for Systematic Reviews and Meta-Analyses
QC: quality criterion
RQ: research question
VR: virtual reality
XR: extended reality

## Data Availability

All data generated or analyzed during this study are included in this published article (and its multimedia appendices).
